# Reduced Susceptibility to Interference in the Consolidation of Motor Memory before Adolescence

**DOI:** 10.1371/journal.pone.0000240

**Published:** 2007-02-28

**Authors:** Shoshi Dorfberger, Esther Adi-Japha, Avi Karni

**Affiliations:** 1 The Laboratory for Functional Brain Imaging and Learning Research, The Brain Behavior Research Center, University of Haifa, Haifa, Israel; 2 School of Education, Bar Ilan University, Ramat-Gan, Israel; Harvard Medical School, United States of America

## Abstract

Are children superior to adults in consolidating procedural memory? This notion has been tied to “critical,” early life periods of increased brain plasticity. Here, using a motor sequence learning task, we show, in experiment 1, that a) the rate of learning during a training session, b) the gains accrued, without additional practice, within a 24 hours post-training interval (delayed consolidation gains), and c) the long-term retention of these gains, were as effective in 9, 12 and 17-year-olds and comparable to those reported for adults. However, a follow-up experiment showed that the establishment of a memory trace for the trained sequence of movements was significantly more susceptible to interference by a subsequent motor learning experience (practicing a reversed movement sequence) in the 17-year-olds compared to the 9 and 12-year-olds. Unlike the 17-year-olds, the younger age-groups showed significant delayed gains even after interference training. Altogether, our results indicate the existence of an effective consolidation phase in motor learning both before and after adolescence, with no childhood advantage in the learning or retention of a motor skill. However, the ability to co-consolidate different, successive motor experiences, demonstrated in both the 9 and 12-year-olds, diminishes after puberty, suggesting that a more selective memory consolidation process takes over from the childhood one. Only the adult consolidation process is gated by a recency effect, and in situations of multiple, clashing, experiences occurring within a short time-interval, adults may less effectively establish in memory experiences superseded by newer ones.

## Introduction

While several lines of evidence indicate that declarative (“what”, explicit) memory undergoes maturation, it is commonly assumed that procedural (“how-to”, implicit) memory, in children, is similar or even superior to that of adults [1]–[4]. The latter notion has been invoked in relation to ‘critical’, early life, periods of increased brain plasticity and skill acquisition [2], [5]–[6] i.e., maturational windows of opportunity wherein neuronal properties are particularly susceptible to shaping by experience [6]–[9]. It has also been invoked in explanations of the larger long-term deficits following brain injury and the less favorable outcome of remediation in adults compared to children [2], [10]. But are children superior to adolescents and young adults in terms of procedural memory consolidation? It was previously shown that, in adults, the evolution of skilled performance often extends beyond the actual training experience. Significant training-dependent gains in performance can appear hours after the termination of training, for example by 24 hours post-training [11]–[18]. It was proposed that these delayed (“off-line”) gains in performance reflect neuronal memory consolidation processes that are triggered by the training experience within the processing stream involved in task performance, but require time, and often sleep, to reach completion [13], [16], [18]. The resultant gains were maintained for weeks and months [12]–[13], [15], [19]. There is, however, a second, time-dependent, behavioural indication for the existence of a latent memory consolidation phase in human skill learning. The retention of training-dependent performance gains on a motor task may be lost or markedly reduced by the introduction of a subsequent training experience, if the latter occurs within up to a few hours after the termination of training on the former task [20]–[23, for a perspective 9]. Presumably, within this interval, ongoing neuronal processes subserving memory retention can be reversed, or interfered with, but once completed, become immune to interference (“stabilization”) [9], [24]. The notion of childhood superiority in procedural learning was tested in Experiment 1 which showed no advantage for children before the onset of adolescence in either within-session or between-session (consolidation phase) gains, nor in long-term retention. The results, however, provided clear evidence for the existence of an effective consolidation phase in motor memory before the onset of adolescence. Experiment 2 tested the possibility that “childhood advantage” in procedural learning reflects a maturational difference in the susceptibility of the learning to interference by a subsequent training experience, and not superior learning and memory per-se.

## Results

**Figure 1:**
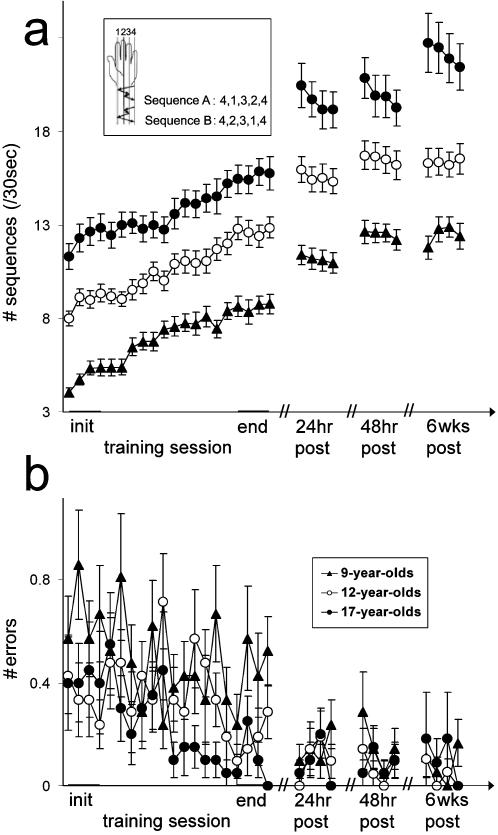
Within-session and between-sessions gains in performance in the 3 age-groups. Inset: the two finger-to-thumb opposition movement sequences used in the study. (a) Mean number of correct sequences, and (b) mean number of errors performed in each test interval (block) during the training session and at 24, 48 hours and 6 weeks post training. Bars–standard error.

In all three age-groups tested in Experiment 1 of the current study (Figure 1) there were, as well as significant within-session improvements, robust delayed (between-session) gains in the performance of the trained sequence of movements as expressed at 24 and 48 hours post-training compared to the performance at the end of the training session. Comparisons of the three age-groups' performance at the four assessment time points was made using a 3 (age-group; 9, 12, 17-year-olds, as between-subject factor) ×4 (time point; init, end, 24 hours and 48 hours–the initial four blocks, final four blocks of the session and the four blocks at 24 hours and at 48 hours post-training, respectively; as within-subject factor) ANOVA. This showed a main effect for time-point, for both the number of sequences (increased speed) and (a reduction in) the number of errors (accuracy) (*F*_(2,177)_ = 273.93, *P*<.001; *F*_(2,177)_ = 18.09, *P*<.001; speed and accuracy respectively) and a significant age effect (*F*_(2,59)_ = 42.1, *P*<.001; *F*_(2,59)_ = 6.01, *P*<.001; speed and accuracy respectively) with no significant interaction. As Figure 1 clearly shows, all three age-groups showed significant within-session gains (comparison of init and end) (*F*_(1,59)_ = 171.15, *P*<.001; *F*_(1,59)_ = 12.18, *P*<.001, speed and accuracy respectively) as well as significant delayed gains, i.e., gains evolving after the termination of the session (comparison of end and 24 hours post-training) (*F*_(1,59)_ = 156.27, *P*<.001; *F*_(1,59)_ = 5.07, *P*<.05, speed and accuracy respectively). This improvement in both speed and accuracy suggests no speed-accuracy trade-off [25], a pattern of results that was proposed as a hallmark for skill acquisition [26]. Moreover, the gains attained by the 48 hours post-training test were completely retained over an interval of 6 weeks, with no additional training during this interval, in all three age-groups; indeed there was a trend for improvement in an (ANOVA comparing 48 hours post-practice performance to performance at 6 weeks post-training; *F*_(1,45)_ = 3.02, *P* = .09).

The initial performance on the task was age dependent, with performance speed and accuracy increasing with age (Figure 1). The performance gains accrued during training, and at 24 hours and 48 hours, as well as at 6 weeks post-training did not differ significantly between the three age-groups. The absolute delayed gains in performance speed, at 24 hours post-training, were largest in the 17-year-olds (2.54±1.47, 2.90±1.66, 4±2.66 additional sequences per block for the 9, 12 and 17-year-olds respectively; *F*_(2,59)_ = 2.96, *P*<.06). Relative to their initial performance, however, the youngest group showed the largest improvement by 24 and 48 hours post-training (by 57%, 34% and 33% at 24 hours post training (*F*_(2,59)_ = 4.89, *P*<.01); and by 30%, 11%, and 4% during the subsequent 24 hours interval (*F*_(2,59)_ = 9.51, *P*<.001) for the 9, 12 and 17-year-olds, respectively).

The 17-year-olds were able to complete twice as many sequences during the training session compared to the 9-year-olds (274.6±56.09 and 139±34.1 sequence iterations, respectively). To rule out the possibility that the delayed gains of the older age-group were dependent on the more intensive experience, an additional group (Group 4) of twelve 17-year-olds (7 girls and 5 boys, M = 16.47 years, range = 16.33–16.66) were given only 11 training blocks (mean of 136.33±26.71 repetitions) in the training session. The results revealed significant delayed gains (12.62±2.4, 15.64±3.11, mean number of sequences at the end of the training session and at 24 hours post-training, respectively; *t*_(11)_ = 5.5, *P*<.001). Moreover, these delayed gains in performance speed were comparable to those accrued in the 17-year-olds trained with the original protocol (Experiment 1, group 3) (*t*_(30)_ = 1.11, *P* = .27).

**Figure 2:**
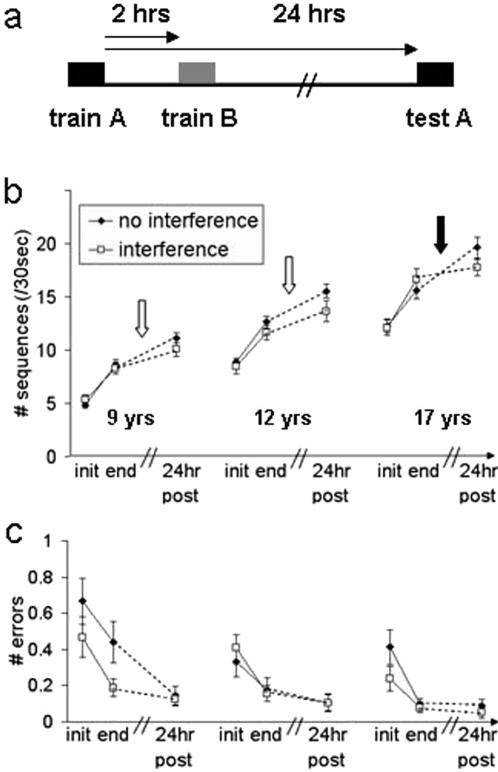
Age dependent effect of post-training interference. (a) Speed and (b) accuracy gains with (♦) and without (□) interference training in the three age-groups. Interference training was given at 2 hours after the termination of the initial training session. Average performance in the initial (init) and the final (end) four blocks of the initial training session, and in four consecutive blocks at 24 hours post-training (24hr post) is shown. Bars–standard error; black arrow - significant interaction; white arrows - no interaction (significant gains in both experiments). Comparison between the three experimental groups' performance at the end of training and at 24 hours post-training in the two experiments, without and with interference (repeated measures ANOVA) showed a significant main effect for time-point for both the number of sequences (speed) and the number of errors (accuracy) (speed: *F*_(1,110)_ = 152.06, *P*<.001 accuracy: *F*_(1,110)_ = 7.11, *P*<.01) and for age (speed: *F*_(2,110)_ = 60.01, *P*<.001 accuracy: *F*_(2,110)_ = 6.89, *P*<.001). The only significant interaction (age-group×assessment time×experiment) was for performance speed (*F*_(2,110)_ = 6.82, *P*<.05) with the 17-year-olds showing less improvement in the interference condition. There was no significant difference between the two experiments for the end time-point in the 17-year-olds (*t*_(37)_ = 0.81, *P* = .42). An analysis of variance for repeated measures, conducted for each age-group separately (with time-points as within-subject factor and age-group and experiment as between-subject factors), showed a significant difference in between-session gains accrued for the initially trained sequence (task A) between the two experiments only in the 17-year-olds (interaction of time-point×experiment, *F*_(1,37)_ = 10.62, *P*<.001). The 9 and 12-year-olds improved to a similar degree with and without interference (no interaction of time-point×experiment, *F*_(1,35)_ = 2.89, *P* = .1; *F*_(1,38_ = 2.21, *P* = .15, 9 and 12-year-olds respectively).

In Experiment 2, the ability of 9, 12 and 17-year-olds to consolidate the training induced gains was tested in an interference paradigm of the form task A–task B–test A (Figure 2a). Separate repeated measures ANOVA with time-points (init, end) as within-subject factor and age-group as between-subject factors were conducted for the interference task (task B). There were significant within-session gains in speed (*F*_(1,51)_ = 81.30, *P*<.001) and in accuracy *(F*_(1,51)_ = 27.26, *P*<.001). There were also significant delayed gains in all three age groups on task B when tested 24 hours post-training, compared to the performance level at the end of the interference training session (ANOVA with time-points (end, 24 hours post training) as within-subject factor and age-group as between-subject factors *F*_(1,51)_ = 65.2, *p*<.001), with no significant interaction of age-group and time-points (*F*_(2,51)_ = 1.67, *p* = .2). In addition, there was a trend for improvement in accuracy (*F*_(1,51)_ = 3.22, *P* = .08) across three age-groups, with no significant interaction of age-group and time-points (*F*_(2,51)_ = .98, *p* = .4).

Figure 2 depicts the mean speed and accuracy for the initially trained movement sequence (task A), in Experiments 1 and 2, for the three age-groups, within the initial training-session and at 24 hours post-training (between-session gains). The initial performance and the within-session gains, in each age-group, in experiment 2 were not significantly different from those attained in experiment 1, where no interference (no task B) was afforded, in terms of both speed (*F*_(1,110)_ = 0.01, *P* = .96) and accuracy (*F*_(1,110)_ = 1.61, *P* = .21) (Figure 2b,c).

**Figure 3:**
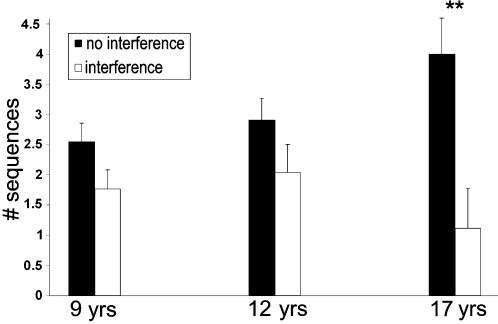
Between-session (delayed) gains with (□) and without (▪) interference training in the three age-groups. The absolute gains in terms of mean number of correct sequences at 24 hours post-training compared to the end of the training session. Bars–standard error. There was a significant interaction of condition by age-group for the mean between-session gains *(F*_(2,110)_ = 6.82, *P*<.05). Independent-samples t-tests conducted for each age group separately showed a significant advantage of the no interference over the interference condition only in the 17-year-olds (*t*_(37)_ = 3.26, *P* = 0.02). There were no significant differences in delayed gains in the two conditions for the 9 and 12-year olds (*t*_(35)_ = 1.7, *P* = 0.1; *t*_(38)_ = 1.49, *P* = 0.002, 9 and 12-year-olds respectively).

Overall, there were significant between-session performance gains in both experiments, for speed (*F*_(1,110)_ = 152.06, *P*<.001) and accuracy (*F*_(1,110)_ = 7.11, *P*<.01) in the initial task (task A). However, there was a significant interaction (age-groups×time-points×experiment) for speed (*F*_(2,110)_ = 6.82, *P*<.05) because, surprisingly, both the 9 and the 12-year-olds showed robust delayed gains in performance of task A even in Experiment 2 (Figure 2b). Only the 17-year-olds showed a significant susceptibility to interference by task B, as expected, i.e., reduced performance on task A at 24 hours post-training relative to the end of the session, in Experiment 2 (interference) compared to Experiment 1 (no interference). The delayed gains (additional sequences per block) in the two condition (with and without interference) were: without interference: 2.55±1.47, 2.90±1.66, 4.00±2.66; with interference: 1.77±1.27, 2.04±2.02, 1.12±2.86 in the 9, 12 and 17-year-olds respectively (Figure 3). Thus, while significant between-sessions improvements in speed occurred in the two younger age-groups, even when interference training was present (*t*_(15)_ = 5.59, *P*<.001, *t*_(18)_ = 4.39, *P*<.001 for the 9-year-olds and 12-year-olds, respectively) the 17-year-olds improved between-sessions only in the absence of interference (*t*_(19)_ = 6.74, *P*<.001), whereas in the presence of interference no improvement occurred ( *t*_(18)_ = 1.70, *P* = .11).

An additional repeated measures ANOVA, with time-point as within-subject factors and experiment as between-subject factor was run for each age-group separately, to test whether the above significant interaction of time-points×experiment in the 17-year-olds was related to the within-session gains in the two experiments (Figure 2b). This analysis showed no significant interaction in any of the three age-groups, indicating that only the delayed gains were affected, in the 17-year-olds, by interference training (*F*_(1,35)_ = 2.66, *P* = .11; *F*_(1,38)_ = 1.05, *P* = .31; *F*_(1,37)_ = 2.11, *P* = .15, for the 9, 12 and 17-year-olds respectively).

In the 17-year-olds the higher the absolute performance achieved in the interfering sequence (B) the lower the performance gains achieved for sequence A at 24 hours post-training (r = −.53, p<.05). In the 12-year-olds however, the correlations were positive, the higher the performance achieved on the interference sequence the larger the gains in sequence A at 24 hours post-training (r = .56, p<.05). Nevertheless, the within-session gains for the interference sequence (B) were not significantly correlated with the delayed gains on the initial sequence (A) in both the 17 and 12-year-olds (r = −.39, p = .1; r = .001, p = 1; 17 and 12-year-olds respectively).

There was an overall improvement in accuracy within-session (main effect for time-point, *F*_(1,51)_ = 23.06, *P*<.001) and a trend for improvement in the between-session accuracy (*F*_ (1,51)_ = 3.25, *P* = .07) in the interference groups, with very few errors at the termination of the session and at 24 hours post-training (mean of 0.14 and 0.09 errors, respectively) and no age-group differences (main effect for age-group) in either the within-session (*F*_(2,51)_ = 0.60, *P* = .55) or the between-session gains (*F*_(2,51)_ = .17, *P* = .84).

## Discussion

The two experiments reported here address two aspects of human memory consolidation in motor skill learning - the evolution of delayed (“off-line” learning) performance gains (Experiment 1) and the susceptibility to interference (Experiment 2) - in children and adolescents, before and after the onset of adolescence. The results of Experiment 1 showed that, in children as well as in adolescents, training on a given sequence of movements resulted not only in significant gains concurrent with the training experience (within-session gains), but also in additional, robust, between-session gains as expressed at 24 hours after the termination of the training experience. This is a first demonstration of “off-line” improvement in children, indicating the existence of an effective consolidation phase in motor memory before the onset of adolescence, in clear similarity to the results recently reported in adults using the same task and a similar training protocol [15].

The younger age-group showed no advantage over the older ones in either within or between-session gains. Moreover, the absolute between-session gains were largest in the 17-year-olds. Only relative to their poor initial performance, the youngest age-group showed superior gains compared to that of adolescents. Moreover, in all three age-groups tested, these gains were fully maintained across an interval of 6 weeks. Taken together, our results suggest that the rate of learning during a training session, the additional, delayed, gains accrued within the 24 hours post-training interval (“off-line” gains), and the long-term retention of these gains, were as effective in 9, 12 and 17-year-olds and comparable to those reported for adults. Thus, the learning and retention of the finger opposition sequence by children (pre-puberty) was not superior to that of young adults.

The results of Experiment 2 provide, however, for the first time, an indication for an age-dependent divergence in human motor learning. Both the 9 and 12-year-olds, but not the 17-year-olds showed large, significant, delayed gains in the performance of the initially trained sequence even given a subsequent interference experience. Thus, only the 17-year-olds showed the previously described [20], [27] adult pattern of interference. Motor memory consolidation, in the 9 and 12-year-olds, was significantly less susceptible to interference by a subsequent training experience compared to the older age groups. Altogether, our results indicate that the stabilization of the training experience into long-term memory may be qualitatively different before and after adolescence.

One cannot rule out the possibility that the interference training experience in the younger age groups was less effective than the one afforded in the corresponding interval for the 17-year-olds. However, our results showed that in all three age groups the interference training resulted in significant and comparable delayed gains, and moreover, that the within-session performance gains for the interference sequence (B) were not significantly (negatively) correlated with the delayed gains achieved for the initial sequence (A) in both the 17 and 12-year-olds.

The current results raise the possibility that a less selective memory consolidation process, present in 9 and 12-year-olds, may be substituted by or modified to, a more selective one, after puberty. Thus the latter, adult, process may be more strongly gated by a recency effect and in situations of multiple, differing experiences, occurring within a relatively short time interval, may less effectively consolidate preceding experiences if superseded by newer ones [20], [22], [27]–[28]. It may be the case that memory consolidation processes proceed at a much faster rate, and memory stabilization is attained much earlier, in children compared to 17-year-olds and adults. Additional studies, in children of different age-groups, are needed to essay the time-course of and the conditions for, the evolution of delayed gains [for review], [ 16]–[18] as well as the time-course of the interference effects [20]. For example, it is not yet known whether in children, as in adults, time in sleep is necessary for the former but not the latter effects [15], [22], [27], [29]–[30] although evidence suggests that sleep may protect memories from subsequent interference in adults as shown in the study of declarative memory [31] as well as in the finger opposition sequence task [32]. The structure of sleep undergoes substantial changes during puberty [33] and this may constitute a possible substrate for age-dependent differences in memory consolidation.

Recently, the notion of “competitive maintenance”, referring to a competition for transcription and protein synthesis related factors within neurons participating in the representation of two independent experiences, following each other within the time-window of consolidation was proposed as a candidate substrate underlying the interference phenomenon [24]. The current results are in line with the notion that the neuronal substrates for such “competitive maintenance” may be set up, or fully mature, only during puberty. Alternatively, given that interference occurs only between tasks that overlap at some common level of neural processing [e.g.], [20,34], it may be the case that in the younger age-groups, the two movement sequences (the initially and the subsequently trained ones) although composed of the same movements, share significantly less of a common neural substrate in children [28], [35]. A third, related, possibility is that the training with one or the other sequence relates to different parameters of the experience in children and adults and thus results in changes in different representations of the movements [35] before and after puberty. For example it may be the case that, in children, the experience of training on any sequence of finger opposition movements may affect the performance of the individual component movements rather than the syntactic rule which has been implicated in the learning of the task by adults [13], [15], [36]. In children, thus, training on one sequence followed by the other would result in enhancement of the training experience as both sequences are composed of the same component movements. Both these notions entail the expectation that the specificity of the learning in children will differ from the one characterizing adult learning [15], [20], [37]. Specifically, that the knowledge retained from a given training experience will be more susceptible to transfer to novel conditions (e.g., a novel sequence) in children compared to adults [11], [13], [15]–[16], [20], [23], [27], [38].

Altogether, our results show that children before the onset of adolescence show no advantage in the acquisition and retention of a given sequence of movements compared to young adults. Moreover, our results show that, in children, motor performance continues to improve in the post-training interval, indicating the existence of a memory consolidation phase, similar to the one recently described in adults. However, our results also suggest that rather than having less effective motor skill learning or memory consolidation processes per-se, adults may be more selective in terms of procedural memory consolidation compared to children, as evidenced by their susceptibility to interference. This may account in part for the discrepancy between the notion of critical periods and maturational windows of opportunity in the acquisition of skills on the one hand and the accumulating evidence for very effective skill learning, both motor and perceptual, in adults, on the other [11]–[13], [15], [17], [19]–[20], [23], [27], [38].

## Materials and Methods

### Participants

Seventy four participants took part in Experiment 1: Group 1–9-year-olds (10 girls and 11 boys, M = 8.55 years, range = 8.24–9.2), Group 2–12-year-olds (10 girls and 11 boys, M = 11.51 years, range = 11.2–12.2), Group 3–17-year-olds (10 girls and 10 boys, M = 16.63 years, range = 16.33–17.5). An additional group of 17-year-olds (7 girls and 5 boys, M = 16.47 years, range = 16.33–16.66) served in the control experiment (Group 4). Fifty four participants took part in Experiment 2: Group 5–9-year-olds (8 girls and 8 boys, M = 8.58 years, range = 8.43–8.81), Group 6–12-year-olds (9 girls and 10 boys, M = 11.874 years, range = 11.5–12.25), Group 7–17-year-olds (9 girls and 10 boys, M = 16.894 years, range = 16.6–17.5). Participants were right-handed, had no medical conditions that could impair fine motor performance, reported at least 6 hours of sleep per night, and had no sleep–wake-cycle disruptions. Inclusion criteria were identical for both experiments: a) a thumb movement rate above 60, 70 or 80 movements in a 30 sec measurement interval (for the 9-year-olds, 12-year-olds and the 17-years-olds, respectively) using a thumb movement counter, and b) 5/5 digits remembered in a forward digit span test. Participants with special finger motor skills (blind typing or keyboard or string instrument playing) were excluded. The experiment was approved by the University of Haifa ethics committee as well as the Ministry of Education, and informed parental consent was obtained.

### The task

The motor task was the finger-to-thumb opposition sequence learning task as previously described [13], [15], [37]. Participants were instructed to oppose the fingers of the left (non-dominant) hand to the thumb in a given 5 movement sequence “as fast and accurately as possible” (Figure 1, inset). Two sequences of equal length and complexity were used, each the reverse of the other. The specific training sequence was randomly assigned. The participants performed the instructed movements while lying supine with the hand positioned on the subject's chest with the elbow flexed, in direct view (palm-facing) of a video camera, to allow recording of all digit finger movements. Visual feedback was not afforded. The training for all age-groups was administered during the morning hours, 9am–12noon.

### Procedure

Experiment 1 included three videotape-recorded sessions in three successive days. In the first session (day 1) each participant underwent training that consisted of 20 blocks each constituting a 30 sec interval. The initiation of each block and it's termination were cued by an auditory signal. Participants were instructed to tap the movement sequence continuously until given the stop signal, and if any error occurred to continue with the task without pause, as smoothly as possible. The breaks between blocks were no longer than 20 sec long. Before each block the participants repeated the assigned sequence three times, freely, as a means for maintaining their attention on the task, and as a practice run. No feedback on any performance measure was provided, but for general encouragement. In the second session (day 2, 24 hours after session 1) and in the third session (day 3, 48 hours post-training) participants were tested in 4 successive blocks identical to the blocks used in the first session. 48 participants (19, 18, 11 from the 9 12 and 17-year-old groups respectively) were tested for retention of the performance gains at 6 weeks post-training. Experiment 2 included three videotape-recorded sessions on two successive days. On day 1, the first session was identical to the first, training, session of experiment 1, but was followed by a second training session, 2 hours later. The latter session was identical to the first, except that the trained sequence was the reverse of the one used in the first session (Figure 1, inset). On the following day, in the third session, participants were tested on 4 blocks of the initially trained sequence using the trained hand.

Two dependent variables were measured and analyzed separately: a) performance speed–the mean number of correct sequences tapped during each block (30 sec interval); b) accuracy–the mean number of sequencing errors (wrong finger opposition order) during each block. Except for age-group and experiment which constituted between-subject factors, all other factors were considered as within-subject factors in the analyses of variance.
